# Flatfishes colonised freshwater environments by acquisition of various DHA biosynthetic pathways

**DOI:** 10.1038/s42003-020-01242-3

**Published:** 2020-09-18

**Authors:** Yoshiyuki Matsushita, Kaho Miyoshi, Naoki Kabeya, Shuwa Sanada, Ryosuke Yazawa, Yutaka Haga, Shuichi Satoh, Yoji Yamamoto, Carlos Augusto Strüssmann, John Adam Luckenbach, Goro Yoshizaki

**Affiliations:** 1 Department of Marine Biosciences, Tokyo University of Marine Science and Technology, 4-5-7 Konan, Minato-ku, Tokyo, 108-8477 Japan; 2 Environmental and Fisheries Sciences Division, Northwest Fisheries Science Center, National Marine Fisheries Service, National Oceanic and Atmospheric Administration, 2725 Montlake Boulevard East, Seattle, WA 98112 USA

**Keywords:** Fatty acids, Ichthyology

## Abstract

The colonisation of freshwater environments by marine fishes has historically been considered a result of adaptation to low osmolality. However, most marine fishes cannot synthesise the physiologically indispensable fatty acid, docosahexaenoic acid (DHA), due to incomplete DHA biosynthetic pathways, which must be adapted to survive in freshwater environments where DHA is poor relative to marine environments. By analysing DHA biosynthetic pathways of one marine and three freshwater-dependent species from the flatfish family Achiridae, we revealed that functions of fatty acid metabolising enzymes have uniquely and independently evolved by multi-functionalisation or neofunctionalisation in each freshwater species, such that every functional combination of the enzymes has converged to generate complete and functional DHA biosynthetic pathways. Our results demonstrate the elaborate patchwork of fatty acid metabolism and the importance of acquiring DHA biosynthetic function in order for fish to cross the nutritional barrier at the mouth of rivers and colonise freshwater environments.

## Abstract (web summary)

Matsushita et al. demonstrate the evolution of DHA biosynthetic mechanisms in four species of flatfish as some of them colonised freshwater environments. Their analyses show independent changes to the biosynthetic pathways as a way to overcome the lack of exogenous DHA that would typically be available from prey in the marine environment.

## Introduction

**Fig. 1:**
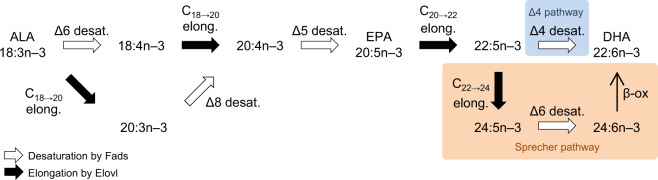
The DHA biosynthetic pathway in teleost fishes. Fatty acids are represented by the *n* − *x* system of nomenclature. Desaturation and elongation steps are indicated by white arrows with the position of the double bond to be introduced and black arrows with carbon chain length, respectively. The two alternative pathways used to synthesise DHA from 22:5*n* − 3 are coloured with orange and blue for the Sprecher pathway and Δ4 pathway, respectively.

Docosahexaenoic acid (DHA; 22:6*n* − 3), an omega-3 long-chain polyunsaturated fatty acid (LC-PUFA), is a crucial fatty acid that supports various functions of the cell membrane as a component of the lipid bilayer, owing to its structural flexibility, and further modulating physiological processes directly or as a precursor of bioactive derivatives^1–4^. It is obtained either as preformed DHA or can be synthesised in most vertebrates from α-linolenic acid (ALA; 18:3*n* − 3; Fig. 1), which is a dietary essential fatty acid^4^. The first step in the DHA biosynthetic pathway is Δ6 desaturation of dietary ALA to 18:4*n* − 3, followed by elongation to 20:4*n* − 3 or, alternatively, elongation of ALA to 20:3*n* − 3, followed by Δ8 desaturation to 20:4*n* − 3. Then, using 20:4*n* − 3 as a substrate, eicosapentaenoic acid (EPA; 20:5*n* − 3) is produced by Δ5 desaturation. In teleost fishes that possess the ability to synthesise DHA, the production of DHA from EPA is achieved by two alternative routes, called the “Sprecher pathway” and “Δ4 pathway”^5–7^. The former consists of two consecutive elongations to produce 24:5*n* − 3, followed by Δ6 desaturation to 24:6*n* − 3 and chain shortening to DHA by β-oxidation at the end (Fig. 1). The latter route is initiated by a single elongation to 22:5*n* − 3, followed by the direct conversion to DHA via Δ4 desaturation. The desaturation and elongation reactions described above are catalysed by fatty acid desaturases (Fads) and elongation of very long-chain fatty acids (Elovl) proteins, respectively. These enzymes have their own substrate specificities to share the pathway and complete DHA biosynthesis by their concerted actions^5,7^.

Spiny-rayed fishes (superorder Acanthopterygii), the largest and most diverse group of fishes, have a limited repertoire of enzymes for DHA biosynthesis^7–9^. In particular, marine Acanthopterygii possess only two enzymes, namely Fads2, which has Δ6/Δ8 desaturase activity^5,10^, and Elovl5, which shows preferential elongase activity towards C_18_ and C_20_ substrates^11^. Moreover, it was previously demonstrated by functional characterisation in a heterologous expression system that Fads2 of marine Acanthopterygii have little or no Δ6 desaturase activity towards 24:5*n* − 3^6^. Therefore, marine Acanthopterygii species strictly require DHA from their diet and develop fatal disorders if they are raised under DHA-deficient conditions^12,13^. A potential cause for the loss of DHA biosynthesis capability is low selective pressure due to the marine food web already being rich in DHA produced by marine microalgae and microbes^12,14^. Hence, marine Acanthopterygii can easily satisfy their DHA requirements by feeding on their natural prey, despite their incomplete DHA biosynthetic pathway^15^. In contrast, the availability of DHA in freshwater (FW) prey is limited^15^, as primary producers in the FW food web are characterised by containing substantial levels of ALA, but are generally poor in DHA^16^. The fact that FW species belonging to Osteoglossomorpha and Otophysi (the oldest living and most diverse FW teleost lineages, respectively^17,18^) possess the capability for DHA biosynthesis^8,19–22^ suggests that dietary DHA obtained in the FW environment is not sufficient for normal development and survival. Still, some lineages of marine Acanthopterygii, which originally and exclusively relied on exogenous DHA, have successfully invaded and colonised FW environments^23,24^. Although the marine-to-FW transition has hitherto been attributed to the substantial adaptation of the osmoregulatory system to overcome the osmotic barrier^25,26^, there is another potential barrier that had to be conquered by Acanthopterygii species moving into the FW environment: the gap between the physiological demand and the dietary supply of DHA.

This study focused on flatfishes (Pleuronectiformes: Acanthopterygii) distributed in marine and FW environments to examine the nutritional barrier created by DHA-deficient prey in FW and the adaptive mechanisms that may allow marine-origin Acanthopterygii to occupy FW habitats. Although this taxonomic order principally consists of marine species, many species from several families migrate to estuaries, which function as nursery grounds, and some even move up into rivers^27,28^. To understand the life histories and optimise the culture conditions of these ecologically and commercially important fishes, their salinity tolerance has been investigated intensively, and many families are categorised as euryhaline^29^. However, there have been no reports of any flatfish species with a complete DHA biosynthetic pathway from ALA^5,30^. Dietary DHA deficiency causes poor growth and survival and developmental abnormalities in marine flatfishes^31^ and hence should result in decreased fitness under natural conditions. Indeed, only 10 out of the 772 species (1.3% of the total diversity) in this order are thought to be FW residents^28^. We consequently hypothesised that this nutritional barrier provides evolutionary pressure that obstructs flatfishes with low-salinity tolerance from attempting to colonise FW environments.

A family of Pleuronectiformes, Achiridae, commonly called American sole, which has expanded its habitat from the sea to rivers^32^, is now distributed across the coastal area of the Americas to the western Amazon. It comprises six genera containing ~35 species, from which six are restricted entirely to FW^28,33^, and the different genera show varying tendencies in terms of habitat preference^33^. Hence, Achiridae is an interesting example of marine-derived Acanthopterygii that can be used to explore the physiological basis for habitat adaptation.

In this study, four Achiridae species were analysed: *Gymnachirus melas* (marine; distributed along the east coast of the USA, eastern Gulf of Mexico, and Bahamas^34^), *Trinectes maculatus* (catadromous; distributed along the eastern coast of North America from Maine (USA) to Mexico^35,36^), *Apionichthys finis* (FW; distributed through the Essequibo and middle and upper Amazon basins^37^), and *Hypoclinemus mentalis* (FW; distributed through the Amazon, Orinoco, and Essequibo basins^38^). First, we examined their migratory histories and phylogenetic relationships to illustrate FW colonisation of Achiridae. Second, the capabilities for DHA biosynthesis in the four species were investigated using brain and hepatic cells isolated from each species, cultured with radiolabelled fatty acid substrates. Third, we conducted functional characterisation of fatty acid metabolising enzymes and revealed their divergent functions, including the first discovery of a trifunctional Δ4Δ5Δ6 Fads2 to our knowledge, giving rise to different, yet fully functional and complete DHA biosynthetic pathways among the three FW species.

## Results and discussion

### Estimating migratory histories and phylogenetic relationships

**Fig. 2:**
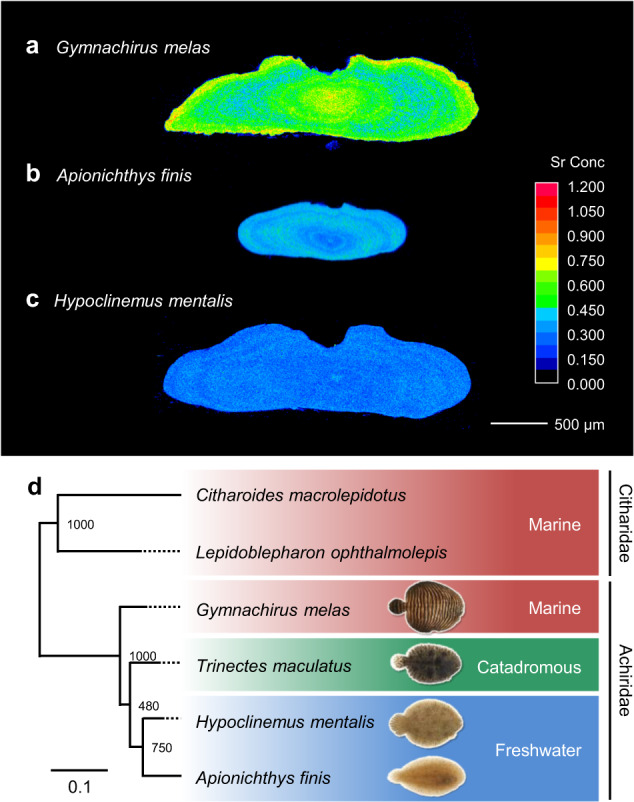
Migratory histories, phylogeny, and habitats support the radiation to freshwater in the lineage of Achiridae. **a**–**c** Representative maps of strontium (Sr) concentration in cross-sections of otoliths from three Achiridae species including a marine species (*G. melas* [*n* = 6]) and two freshwater species (*A. finis* [*n* = 4] and *H*. *mentalis* [*n* = 6]). Scale bar = 500 µm. **d** Phylogenetic tree of Achiridae and Citharidae (as an outgroup) based on the 16S rRNA gene (partial sequence, 958 bp) shown along with their habitats.

We used otolith analysis to examine their environmental life histories, and specifically, to determine whether the so-called FW species had any previous exposure to marine conditions. Otoliths are biominerals present in the inner ears that are responsible for the sense of balance in teleost fishes. They consist predominantly of calcium carbonate and, for some short-lived teleosts, grow in proportion to somatic growth of the individual. Otoliths incorporate trace elements that reflect ambient environmental conditions, such as salinity^39^. We analysed the strontium (Sr) concentration, which is positively correlated to the ambient salinity, in transverse sections of the sagittal otoliths (Fig. 2a–c). In the marine species *G. melas*, the Sr concentration was high in the core and peripheral areas, indicating birth and residence predominantly in seawater (SW) (Fig. 2a). However, these otoliths also showed concentric regions with intermediate to low Sr concentration presumed to reflect transient forays into areas of low to moderate salinity, such as an estuary, often used as nursery grounds in this order^27^. The otoliths of *A. finis* and *H. mentalis* showed a low Sr concentration throughout the otolith (Fig. 2b, c), indicating that these fishes were born in and never left the Amazon, where they were captured. Our results are in agreement with previous reports based on capture sites^33^ and demonstrate that *A*. *finis* and *H*. *mentalis* spend their entire life in FW.

Next, we constructed a molecular phylogenetic tree with the closely related ancestral marine family Citharidae^40^ as an outgroup using 16S rRNA genes (Fig. 2d) to investigate the evolutionary relationships among the four species of Achiridae. The resultant tree, which is consistent with previous reports^41,42^, showed *G. melas* as the first species branching from the lineage leading to *A. finis* and *H. mentalis* via the intermediate *T. maculatus*. Our phylogenetic tree suggests that *G. melas*, which relies heavily on marine habitats, maintains some ancestral physiological features associated with FW adaptation.

The low osmolality of the FW environment is an initial cause of disruption of homoeostasis in marine fishes. To gain insight into when the lineage of Achiridae acquired the capability to cope with hypoosmotic stress, we compared the hypoosmotic tolerance of *G. melas* (Supplementary Fig. 1) to that of the euryhaline *T. maculatus*^35,36,43^ and a stenohaline marine Acanthopterygii, the horse mackerel (*Trachurus japonicus*)^44^. The test was carried out by the gradual replacement of SW with FW in a laboratory tank according to a schedule designed so that *T. maculatus* would survive. In this test, *G. melas* showed remarkable tolerance to survive in 10% SW, whereas all horse mackerel used in the study did not. This suggests that a substantial tolerance to low salinity is shared among Achiridae regardless of their natural habitat.

### Tracing fatty acid metabolism in brain and hepatic cells

**Fig. 3:**
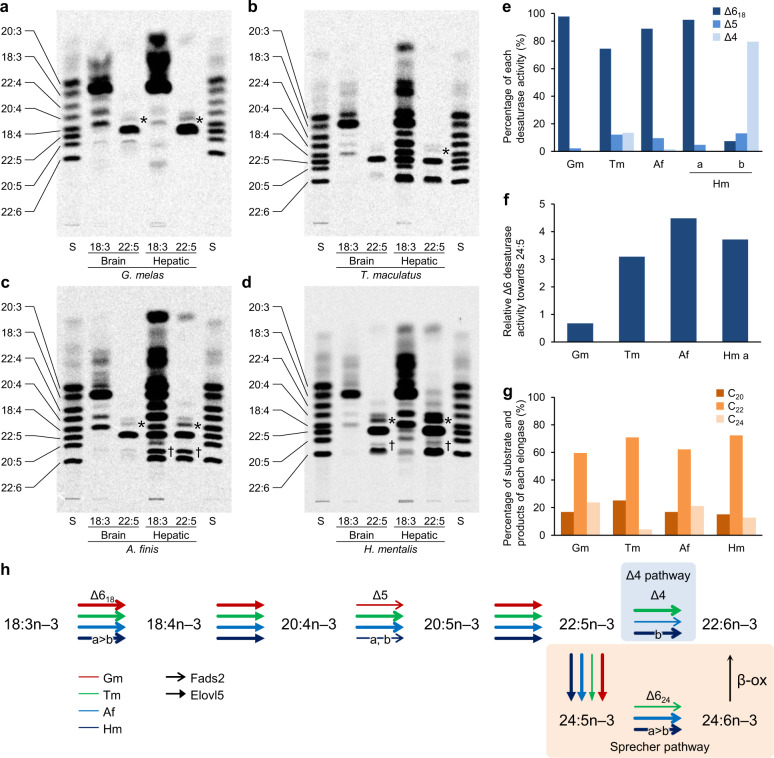
The DHA biosynthetic capabilities were acquired in different ways in three Achiridae species depending on the freshwater environment. **a**–**d** Autoradiography of TLC plate-developed radiolabelled fatty acid methyl esters (FAMEs) prepared from brain and hepatic cells of each Achiridae species cultured with radiolabelled α–linolenic acid (ALA, 18:3*n* − 3) or 22:5*n* − 3. 24:5*n* − 3 and 24:6*n* − 3 are indicated by asterisks and daggers, respectively. S, Standard mixtures of FAME. **e** The ratio among three desaturase activities of fatty acid desaturase 2 (Fads2) isolated from each Achiridae species when the total conversion rates are 100%, characterised by the yeast expression system. Relative conversion rates of Δ6 towards 18:3*n* − 3 (Δ6_18_), Δ5 and Δ4 desaturation are shown as dark blue, blue and light blue bars, respectively. Gm *Gymnachirus melas*; Tm *Trinectes maculatus*; Af *Apionichthys finis;* Hm *Hypoclinemus mentalis*. **f** Relative Δ6 activities towards 24:5*n* − 3 of the Achiridae Fads2 normalised by those of 18:3*n* − 3 when expressed with the zebrafish Elovl2 in yeast to produce 24:5*n* − 3 from 22:5*n* − 3. **g** The ratio of eicosapentaenoic acid (EPA, 20:5*n* − 3, dark orange), 22:5*n* − 3 (orange) and 24:5*n* − 3 (light orange) extracted from yeast cultured with EPA as the substrate and expressed elongation of very long-chain fatty acid 5 (Elovl5) isolated from each Achiridae species. **h** The DHA biosynthetic pathway from 18:3*n* − 3 with enzymatic activities of Fads2 and Elovl5 isolated from each Achiridae species estimated by the radiolabelled metabolites in the cell culture and fatty acid profiles of the yeast expressing recombinant enzymes. Each colour of arrow shows the activity of enzymes isolated from different species (red: *Gymnachirus melas*, green: *Trinectes maculatus*, blue: *Apionichthys finis*, navy: *Hypoclinemus mentalis*). Bold and narrow lines indicate high and low activities, respectively.

We then examined the capability of Achiridae to synthesise DHA from ALA to determine whether there are differences that reflect the nutritional environment of each natural habitat (Fig. 3a–d). For each of the four sole species we cultured cells from brain and liver, where Fads and Elovl are highly expressed^7^, with radiolabelled [1-^14^C]ALA or [1-^14^C]22:5*n* − 3. After 40 h of culture, total lipid was extracted from the cells to prepare fatty acid methyl esters (FAMEs), which were then developed with thin-layer chromatography (TLC). Although incorporation of [1-^14^C]ALA and [1-^14^C]22:5*n* − 3 into the cells was detected, no radioactive DHA (22:6*n* − 3) was detected in *G*. *melas* brain or hepatic cells (Fig. 3a). However, we clearly found radioactive DHA along with a series of intermediates in the FAMEs derived from the hepatic cells of the other three soles, which spend a substantial portion of their life cycle in the FW environment (Fig. 3b–d). It is noteworthy that radioactive 24:5*n* − 3 and 24:6*n* − 3, which are the intermediates in the Sprecher pathway (Fig. 1), were detected in both *A*. *finis* and *H. mentalis*, but especially strong in *A*. *finis* (Fig. 3c, d). Moreover, radioactive DHA synthesised in the brain cells was detected only in *H. mentalis* (Fig. 3d). Our data demonstrate that the three FW-dependent species possess the capability to synthesise DHA from ALA, whereas the marine species, *G. melas*, likely does not. Furthermore, the DHA biosynthetic pathways of the three FW-dependent species appeared to differ, particularly in the availability and utilisation of the Sprecher pathway.

### Functional characterisation of Fads2 and Elovl5 enzymes

To investigate the molecular basis causing the above differences in DHA biosynthetic potency, we conducted functional characterisation of the enzymes Fads2 and Elovl5 involved in DHA biosynthesis with a heterologous expression system in yeast (*Saccharomyces cerevisiae*) (Fig. 3e–g; Supplementary Figs. 2–5 and Table 1a–c)^45^. The Fads2 isolated from *G. melas* showed Δ6 activity towards ALA (Δ6_18:3*n*−3_), low Δ5, and no Δ4 desaturase activities (Fig. 3e). In addition, its Δ6 activity towards 24:5*n* − 3 (Δ6_24:5*n*−3_) was the lowest among the four sole species (Fig. 3f). The *G. melas* Elovl5 exhibited activity towards C_18_ to C_22_ substrates (Supplementary Table 1a) and production of 22:5*n* − 3 and 24:5*n* − 3 from EPA (20:5*n* − 3, Fig. 3g). In the DHA biosynthesis assay using *G. melas* cells, we observed only EPA biosynthesis from ALA in the brain cells and 24:5*n* − 3 biosynthesis from 22:5*n* − 3 in both the brain and hepatic cells (Fig. 3a). Therefore, taken altogether, we concluded that *G. melas* is incapable of DHA biosynthesis due to insufficient activity of Fads2 to desaturate 22:5*n* − 3 or 24:5*n* − 3 (Fig. 3h, red arrows).

In contrast, the Fads2 isolated from *T. maculatus* showed desaturase activity for the Δ6, Δ5 and Δ4 pathways (Fig. 3e, f). This conspicuous trifunctionality has not been reported in any other front-end desaturase known to date^7,10,46^. The Elovl5 showed elongase activities towards C_18_ to C_22_ substrates (Supplementary Table 1a), although the production of 24:5*n* − 3 from EPA was relatively low (Fig. 3g), which is consistent with the weak radioactivity of 24:5*n* − 3 detected in the DHA biosynthesis assay (Fig. 3b). We concluded that *T. maculatus* synthesises DHA from ALA via the Δ4 pathway with the trifunctionalised Fads2 as a key enzyme in this process (Fig. 3h, green arrows).

The Fads2 isolated from *A. finis* showed Δ6_18:3*n*−3_, Δ5, and low Δ4 activities (Fig. 3e) and performed Δ6_24:5*n*−3_ desaturation at the highest efficiency among the four species (Fig. 3f). The Elovl5 showed relatively high production of 24:5*n* − 3 from EPA (Fig. 3g), in addition to the conversion of C_18_ and C_20_ substrates (Supplementary Table 1a). The results clearly supported the detection of radioactive C_24_ fatty acids in the DHA biosynthesis assay (Fig. 3c), and we therefore concluded that *A. finis* conducts DHA biosynthesis from ALA with the enzymes reinforced to drive the Sprecher pathway (Fig. 3h, blue arrows), which is weak or absent in the marine Acanthopterygii.

In the case of *H. mentalis*, we isolated two *fads2* genes sharing 98% identity at the deduced amino acid level and considered that the gene differentiated into paralogues after speciation because they formed a single branch in the molecular phylogenetic tree of Achiridae Fads2 (Supplementary Fig. 6). The two Fads2, namely Fads2a and Fads2b, showed distinct activities, catalysing Δ6_18:3*n*−3_ and Δ4 desaturation, respectively, while also showing Δ5 and Δ6_24:5*n*−3_ activity (Fig. 3e, f; Supplementary Table 1b, c). The Elovl5 performed elongations of C_18_ to C_22_ substrates (Supplementary Table 1a) and production of 22:5*n* − 3 and 24:5*n* − 3 from EPA (Fig. 3g). These activities did not conflict with the moderate radioactivities of the C_24_ fatty acids compared to those of *A. finis* in the DHA biosynthesis assay (Fig. 3d). We concluded that *H. mentalis* synthesises DHA from ALA via both the Δ4 and Sprecher pathways, with the duplicated Fads2 specialised by neofunctionalisation to share the desaturation steps (Fig. 3h, navy).

### Exploring the molecular basis of Fads2 diversification

**Fig. 4:**
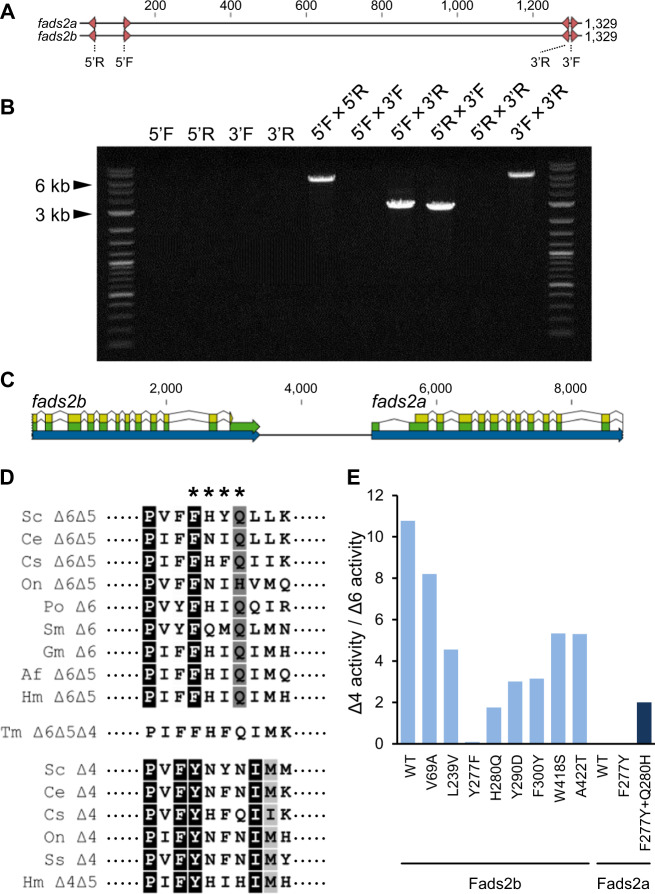
The two *fads2* paralogues of *H. mentalis* were tandemly duplicated in the genome, and their functional differentiation can be determined by two amino acid substitutions. **a** Four primers (5′F, 5′R, 3′F and 3′R) were designed to anneal to conserved regions near both ends of the coding sequences (CDS) of the two *fads2* genes to analyse their genomic loci. **b** Representative electrophoretic image of genomic PCR products of three individuals by the four primers used independently or in pairs. Major bands were amplified by the four combinations of the primers (5F × 5R, 5F × 3R, 5R × 3F and 3F × 3R). **c**, Genomic loci of the two *fads2* genes were predicted by the distribution of the primers and sequencing analysis of the PCR products derived from one representative individual. Regions of the genes, exons and CDS were annotated by blue, green and yellow arrows, respectively. **d** The amino acid sequence alignment of the key residues (indicated by asterisks) for determining the regioselectivity of Fads2 isolated from Achiridae and several teleosts shown with their corresponding desaturase activity characterised in this study (Achiridae species) or reviewed previously (other species)^10^. Sc *Siganus canaliculatus*; Ce *Chirostoma estor;* Cs *Channa striata*; On *Oreochromis niloticus*; Po *Paralichthys olivaceus;* Sm *Scophthalmus maximus;* Ss *Solea senegalensis*. **e** The ratio of Δ4 activity to Δ6 activity towards 18:3*n* − 3 of Fads2a, Fads2b and ten mutant Fads2 carrying substituted residue(s) that differed between the two Fads2 of *H. mentalis*.

To further investigate the origin of the paralogous Fads2 of *H. mentalis*, we conducted PCR with four primers designed to map their genomic loci (Fig. 4a, b). The results demonstrated that they were located tandemly and exhibited conserved exon–intron boundaries (Fig. 4c), suggesting that gene duplication through unequal crossing-over occurred in this lineage^47^. We then analysed the relationship between the remarkable identity and divergent functions of the duplicated Fads2. They showed eight substitutions along their 442 amino acids (Supplementary Fig. 7), with two of them located in the motif related to substrate preference, as demonstrated in previous studies (Fig. 4d)^6,48^. We substituted each of the eight amino acids of Fads2b with those of Fads2a by site-directed mutagenesis, and, indeed, the mutant Fads2b lost preference of Δ4 desaturation by altering Y277 (Fig. 4e; Supplementary Table 1d), which is well conserved in the other Δ4Fads2 found in teleost fishes (Fig. 4d). On the other hand, the mutant Fads2a with Y277 introduced showed no Δ4 activity, while that carrying both F277Y and Q280H in the motif acquired a preference for Δ4 desaturation (Fig. 4e, Supplementary Table 1d). Our results suggest that a few point mutations arose and directed the gene towards neofunctionalisation. Although duplications and neofunctionalisation of *fads2* genes are known in several other Acanthopterygii that are herbivorous, diadromous, or FW species^5,7^, our finding in *H. mentalis* provides insight into the evolution of adaptive enzymes generated in the recent past, standing out from the other species in regard to the identity of paralogues. However, the trifunctional Fads2 of *T. maculatus* strays from the rule of the motif because it showed F280 conserved in the corresponding motif in Δ6 Fads2 (Fig. 4d) despite its Δ4 desaturase activity (Fig. 3e; Supplementary Table 1b). It seems that the unique mutations in other regions are responsible for the exceptional function of the enzyme in this species, while the structural basis is still unknown.

**Fig. 5:**
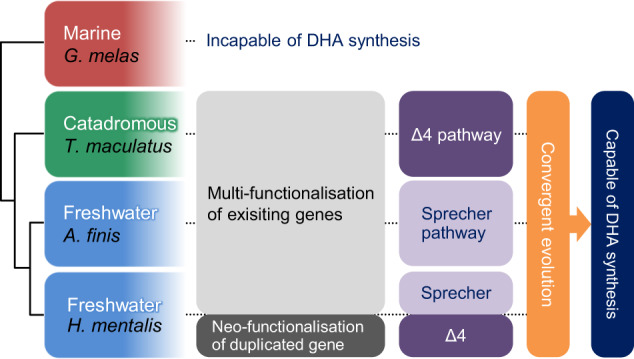
Convergent evolution towards acquiring the capability to synthesise DHA occurred in every sole species that inhabits the freshwater environment. The marine species *G. melas* is unable to synthesise DHA due to the absence or inefficiency of Fads2 activities to desaturate at the Δ4 position or Δ6 position of 24:5*n* − 3. The catadromous species *T. maculatus* has a multi-functionalised Fads2, which alone can catalyse Δ6, Δ5 and Δ4 desaturations to drive the Δ4 pathway. *A. finis* has reinforced Fads2 and Elovl5, which can catalyse C24 fatty acids more efficiently to drive the Sprecher pathway. *H. mentalis* possesses an additional highly homologous Fads2 (Fads2b) that performs Δ4 desaturation, which occurred via neofunctionalisation following gene duplication.

Our data reveal that independently accumulated mutations invited divergent functions of Fads2, and every combination, with Elovl5 showing different substrate preferences, converged to complement the DHA biosynthetic pathway in each FW-dependent species of the family Achiridae. In other words, the genetic basis of each of these sole species has been utilised for a common outcome, DHA biosynthesis from ALA, and towards this end, every possible means has been implemented during natural selection (Fig. 5). Previously, it was demonstrated that a quantitative increase in the number of copies of *fads2* in the genome contributed to FW colonisation of sticklebacks by overcoming the constraint of low levels of dietary DHA^49^. Our work in Achiridae species highlights the FW nutritional barrier in a different light by demonstrating the qualitative alteration of the fatty acid metabolising enzymes, with or without increases in the copy number of these genes, resulting in a variety of functionally complete DHA biosynthetic pathways. We therefore conclude that acquiring the capability for DHA biosynthesis from ALA, in addition to osmoregulation under low salinity, was a precondition for marine Acanthopterygii leaving the cradle filled with DHA and colonising FW environments.

## Methods

### Fish

*G. melas* (12 individuals, standard length (SL) 90.7 ± 2.0 mm, body weight (BW) 17.7 ± 1.1 g) captured in the western Atlantic Ocean were purchased from Quality Marine (Los Angeles, CA). *T. japonicus* (4 individuals, SL 126.3 ± 3.8 mm, BW 37.0 ± 4.9 g) were caught in Tateyama Bay, Chiba, Japan. These two species were maintained in recirculating tanks with artificial SW (33‰, 837 ± 1.2 mOsmol kg^−1^; Tetra Marin Salt Pro, Spectrum Brands Japan) at 24 °C and fed with the polychaete worm *Perinereis aibuhitensis* until use. *T. maculatus* (26 individuals, SL 32.8 ± 2.1 mm, BW 1.3 ± 0.3 g) were purchased from Ishi-to-Izumi (Tokyo, Japan), and *A. finis* (13 individuals, SL 52.5 ± 3.9 mm, BW 2.0 ± 0.7 g) and *H. mentalis* (3 individuals, 129.7 ± 2.9 mm, BW 55.7 ± 6.6 g) were purchased from Ishi-to-Izumi or Kamihata Fish Industry (Hyogo, Japan). These three species were maintained in recirculating tanks with FW at 26 °C and fed with frozen blood worms (larvae of Chironomidae), live sludge worms (*Tubifex tubifex*), or medaka (*Oryzias latipes*). Zebrafish (*Danio rerio*) were maintained as described in a previous study^50^. All experiments were performed in accordance with the guidelines for the care and use of laboratory animals of the Tokyo University of Marine Science and Technology.

### Phylogenetic analysis based on the 16S rRNA gene

Genomic DNA was extracted from the caudal fins of the four sole species using the Gentra Puregene Tissue Kit (QIAGEN). The partial fragments of the 16S rRNA gene were PCR amplified using primers designed based on the orthologues in Achiridae collected from the GenBank database to anneal to conserved regions (Supplementary Data 1, 2) with PrimeSTAR Max DNA polymerase (Takara Bio). All PCR conditions in this research are summarised in Supplementary Data 3. The PCR products were purified with the FastGene Gel/PCR Extraction Kit (Nippon Genetics) and cloned into the pGEM T-Easy Vector (Promega) after adding 3′ adenosine overhangs with TaKaRa Taq (Takara Bio). The cloned PCR fragments were extracted using the FastGene Plasmid Mini Kit (Nippon Genetics) and sequenced using the BigDye Terminator v3.1 Cycle Sequencing Kit and ABI PRISM 3100-Avant Genetic Analyzer (Thermo Fisher Scientific). The resulting sequences were assembled using CLC Main Workbench ver 6.7.1 (QIAGEN) and aligned with the genes from *Citharoides macrolepidotus* (AP014588) and *Lepidoblepharon ophthalmolepis* (KJ433560), which belong to Citharidae, an ancestral family of Pleuronectiformes, using MAFFT v7.222 with the L-INS-i method^51,52^. After trimming gaps automatically using trimAl^53^, a phylogenetic tree was constructed from the alignment made up of 960 columns by the maximum likelihood method using PhyML ver 3.0 server^54^ with Smart Model Selection resulting in GTR + G + I, and the number of bootstrap replicates was set to 1000. The resulting tree was visualised in FigTree ver 1.4.3, available at http://tree.bio.ed.ac.uk/software/figtree/.

### Estimating migratory history using otoliths

The Sr profiles in sagittal otoliths were analysed by electron probe microanalysis (EPMA) to obtain information on the environmental (salinity) history of the fishes. Otoliths were extracted from each individual, cleaned, dried, and embedded in UV-cured resin (Tama-koubou, Kanagawa, Japan)^55^. Each otolith was then ground transversally from the postrostrum side using a series of 120–4000 grid abrasive paper on a grinder/polisher (Doctor-Lap ML-182, Maruto Instruments, Tokyo, Japan) to obtain a transversal section showing all preformed layers including the core region formed at birth. After course grinding, otoliths were further polished to a mirror-finish with 1 µm diamond paste on a polisher (Labopol-4, Struers), cleaned and rinsed with deionised water prior and dried. For EPMA, specimens were platinum/palladium-coated with an ion-sputter (E-1030, Hitachi High-Technologies Corporation) and analysed with an electron probe microanalyser (JXA-8230, JEOL) using Sr titanate (SrTiO3) as a reference standard. Maps of Sr concentration were generated with a focused beam set to 15 kV of accelerating voltage and 500 nA of beam current, with a pixel size of 2 µm and a dwell time of 40 ms.

### Molecular cloning of *fads2*, *elovl5* and *elovl2* genes

Total RNA was isolated from the brains and livers using ISOGEN reagent (Nippon Gene). After trace genomic DNA was eliminated with RQ1 RNase-Free DNase (Promega), the first-strand complementary DNA (cDNA) was synthesised using Ready-To-Go You-Prime First-Strand Beads (GE Healthcare) with the oligo (dT) primer (Supplementary Data 2) following the manufacturer’s recommendations. The first fragment amplifications of *fads2*- and *elovl5*-like cDNA from *H. mentalis* and *T. maculatus* were carried out using the degenerate primers shown in Supplementary Data 2, which were designed based on highly conserved regions of *fads2* and *elovl5* orthologues from several species of Pleuronectiformes (Supplementary Data 1, 2) with TaKaRa Ex Taq polymerase (Takara Bio). To amplify the 5′ and 3′ ends of the cDNA, the rapid amplification of the cDNA ends (RACE) of both *fads2* and *elovl5*-like cDNA from the two species was performed using the GeneRacer Kit (Thermo Fisher Scientific) and the DNA polymerases, PrimeSTAR HS DNA polymerase and Tks Gflex DNA polymerase (Takara Bio). The gene-specific primers for 5′ and 3′ RACE are shown in Supplementary Data 2. The purification, subcloning and sequencing of each PCR product was performed as described above without an A-tailing reaction. The resultant sequences were assembled to produce the full open reading frame (ORF) using CLC Main Workbench ver 6.7.1 (QIAGEN). The cDNA, including full ORFs, was PCR amplified using gene-specific primers that were annealed to the 5′ and 3′ untranslated regions (UTR) with PrimeSTAR Max DNA polymerase. The *fads2*- and *elovl5*-like cDNAs from *G. melas* and *A. finis* were isolated using primers designed to anneal to the conserved regions in the 5′ or 3′ UTR of the two genes from the above two species (Supplementary Data 2). The alignment of the deduced amino acid sequences of *fads2*- and *elovl5*-like cDNA, with several orthologues from the GenBank database, was conducted by MAFFT ver 7.222 with the L-INS-i method. After trimming gaps automatically using trimAl, a phylogenetic tree was constructed from the alignment made up of 445 columns by the maximum likelihood method using the PhyML ver 3.0 server with Smart Model Selection, resulting in LG + G + I, and the number of bootstrap replicates was set to 1000. The resulting tree was visualised in FigTree and rooted with Chondrichthyes Fads1 sequences.

### Functional characterisation of Fads2 and Elovl5

The cDNAs corresponding to the *fads2* and *elovl5* ORFs of the four sole species and *fads2* and *elovl2* of zebrafish were PCR amplified using primers containing restriction enzyme sites of *Hin*dIII and *Xba*I for *fads2* and *elovl5* or *Hin*dIII and *Kpn*I for *elovl2* with PrimeSTAR Max DNA polymerase (Supplementary Data 2). After gel purification following the method described above, the PCR products were then digested by the corresponding restriction enzymes (*Hin*dIII, *Xba*I, and *Kpn*I; Takara Bio) and cloned into the yeast expression vector pYES2 (Thermo Fisher Scientific) digested by *Hin*dIII and *Xba*I for *fads2* and *elovl5* or pAUR123 (Takara Bio) digested by *Hin*dIII and *Kpn*I for *elovl2*. The mutations in *fads2a* and *fads2b* of *H. mentalis* in pYES2 were introduced by inverse PCR using the PrimeSTAR Mutagenesis Basal Kit (Takara Bio) and primers carrying each substitution shown in Supplementary Data 2. Transformation with pYES2 vectors and the culture condition of INV*Sc*1 yeast (*S. cerevisiae*) (Thermo Fisher Scientific) were described in a previous study^45^. The following fatty acids (Larodan Fine Chemicals) were used as substrates: 18:3*n* − 3, 20:4*n* − 3 and 22:5*n* − 3 for the yeast transformed with *fads2* and 18:4*n* − 3, 20:5*n* − 3 and 22:5*n* − 3 for *elovl5*. After 48 h of culture at 30 °C in the presence of each substrate fatty acid, the yeast cells were collected, washed twice with ice-cold HBSS and lyophilised using a freeze dryer (FDU-1200, Tokyo Rikakikai). FAMEs were prepared from the pellets of yeast and analysed using a gas chromatograph (GC-2025, Shimadzu) equipped with a flame ionisation detector and a silica capillary column (L × I.D. 30 m × 0.32 mm, *d*_f_ 0.25 μm; SUPELCOWAX 10, Merck) as previously described^56^. Enzymatic activities were calculated as the proportion of fatty acid substrate converted to desaturated or elongated products with the following formula: [product area/(product area + substrate area)] × 100 (%).

To analyse the Δ6 activity towards 24:5*n* − 3, we constructed co-expression vectors of Achiridae Fads2 and zebrafish Elovl2 using an In-Fusion HD Cloning Kit (Takara Bio). Isolation of the *elovl2* with PADH1 and TADH1 regions from the above pAUR123 vector as an insert and linearisation of the pYES2 vectors carrying each *fads2* were carried out by the primers designed as described in the manual and PrimeSTAR Max DNA polymerase. The recombinant yeast transformed by the resultant vectors were selected and cultured with 22:5*n* − 3 as described above for 24 h, but without induction of the *fads2* connected to PGAL1 by galactose, to allow the elongation of 22:5*n* − 3 to 24:5*n* − 3 by the *elovl2* connected to PADH1, which is constitutively expressed. Then, 2% galactose was added to the yeast cultures for the induction of *fads2* to allow the desaturation of 24:5*n* − 3 to 24:6*n* − 3, and the cultures were further incubated for 48 h until collection. To standardise and compare the Δ6 activity towards 24:5*n* − 3 in Achiridae Fads2, the yeast were cultured in the presence of 18:3*n* − 3 as control according to the above process. The Δ6 activity towards 24:5*n* − 3 was calculated as described above, considering the areas of 24:5*n* − 3 produced by Elovl2 as the substrate, then divided by the Δ6 activity in the control cultures for standardisation.

### Analysis of the genomic loci of *Hmfads2a* and *Hmfads2b*

Two primer pairs were designed that anneal as described in Fig. 4a, but do not extend across the putative exon–intron junctions predicted from the genomic *fads2* structure of *Cynoglossus semilaevis* (Gene ID: 103380276). Genomic DNA fragments were amplified by PCR using combinations of primers (Supplementary Data 2) with TaKaRa LA Taq and PrimeSTAR GXL DNA polymerase. The products of TaKaRa LA Taq were used to determine the draft sequences. The resultant sequences were combined and annotated according to the CDS and UTR based on the cDNA sequences and those of *C. semilaevis*.

### Tracing fatty acid metabolism in cell culture

The capacity for endogenous PUFA biosynthesis in each species was examined using radiolabelled fatty acid substrates. Brain and hepatic cells were collected from *G. melas*, *T. maculatus*, *A. finis*, and *H. mentalis*. The fishes were anaesthetised with 0.02% (v/v) 2-phenoxyethanol, and their bulbus arteriosus was incised to remove the blood with 10 U/ml heparin sodium in Hanks’ balanced salt solution (HBSS) to prevent the contamination of red blood cells following culture. The isolated livers were minced and incubated with 2 mg/ml collagenase IV (C5138, Sigma) and 150 U/ml DNase I (D5025, Sigma) in HBSS for 4 h at 20 °C. After incubation, the resultant hepatic cell suspensions were filtered and rinsed as described in a previous study^57^. The isolated brains were minced and filtered as described in a previous study^58^.

For the LC-PUFA biosynthesis assays, all cell pellets were resuspended in L-15 medium (Invitrogen) containing 50 µg/ml ampicillin sodium (Wako), 50 µg/ml streptomycin sulfate (Wako) and 50 U/ml benzylpenicillin potassium (Wako). For brain cultures, foetal bovine serum (Gibco) was added to the medium at a 10% concentration. The 2 ml cell suspensions were dispensed into six-well plates (*T. maculatus*: 1.2 × 10^7^ hepatic and 7.0 × 10^6^ brain cells, *A. finis*: 1.2 × 10^7^ and 6.2 × 10^5^ cells), or 5 ml was dispensed into 25 cm^2^ tissue culture flasks (*G. melas*: 2.1 × 10^7^ and 1.8 × 10^7^ cells, *H. mentalis*: 2.1 × 10^7^ and 1.8 × 10^7^ cells). The radiolabelled [1-^14^C] 18:3*n* − 3 or [1-^14^C] 22:5*n* − 3 (American Radiolabeled Chemicals) was conjugated with bovine serum albumin (BSA, fatty acid free, A8806, Sigma) as described in a previous study^57^. The cells were cultured with 3.7 kBq (2 nmol)/ml of the PUFA/BSA complexes at 20 °C for 40 h. The cells were then harvested and centrifuged at 400 × *g* for 2 min to discard the supernatant and rinsed with 5 ml of HBSS containing 1% BSA. Total lipids were extracted from the cell pellets using chloroform/methanol (2:1, v/v) as described in previous studies^59,60^. FAMEs were prepared from evaporated total lipids using a Fatty Acid Methylation Kit (Nacalai Tesque, Kyoto, Japan) and purified using a FAME Purification Kit (Nacalai Tesque) following the manufacturer’s instructions. FAMEs were concentrated in 1 ml of hexane, and 400 µl was applied as 1 cm streaks to TCL (20 cm × 20 cm; Merck) plates, which were pre-immersed in 0.1 mg/ml silver nitrate in acetonitrile for 10 min and activated at 110 °C for 30 min. The plates were developed in toluene/acetonitrile (95:5, v/v) and subjected to autoradiography using imaging plates with an image analyser (FLA-9000, Fujifilm).

The radiolabelled [1-^14^C] fatty acids for the standard mixture were purchased from American Radiolabeled Chemicals (18:3*n* − 3, 20:3*n* − 3, 20:5*n* − 3 and 22:5*n* − 3) or Moravek (22:5*n* − 3 and 22:6*n* − 3). These were mixed to 370 Bq each and methyl-esterified as described above to create 1 ml of standard solution (except 20:4*n* − 3) in hexane. The other standards for intermediate metabolites in the DHA biosynthetic pathway, 20:4*n* − 3 and 24:6*n* − 3, were biosynthesised from 18:4*n* − 3 and 24:5*n* − 3 (American Radiolabeled Chemicals) by the yeast transformed with pYES2 carrying *elovl5* from *Nibea mitsukurii*^56^ and *fads2* from *D. rerio*, respectively, following the above method. The yeast were cultured with 3.7 kBq of the fatty acids and lysed by incubation with 50 µl of 30 mg/ml zymolyase (Nacalai Tesuque, Inc.) at 37 °C for 30 min. The total lipids of these lysates were extracted, methylated, and purified as described above to create 1 ml of metabolite solutions in hexane. The final standard mixture was prepared by mixing 100 µl each of the standard solutions (except 20:4*n* − 3) and the metabolite solution of 18:4*n* − 3. The positions where 24:5*n* − 3 and 24:6*n* − 3 appeared on the TLC plates were confirmed using the metabolite solution of 24:5*n* − 3 (Supplementary Fig. 8).

### Acclimation test for hypoosmolality

To examine survival of *G. melas*, *T. maculatus*, and *T. japonicus* in hypoosomotic condition, individuals of these species were exposed to hypoosmolality by discarding half a tank of water and refilling the tank gradually with FW or diluted SW using syphon effect for around 12 h according to the following schedule over 12 days: 50%, 40%, 30%, 20%, and 10% SW for 2 days each. After acclimation to 10% SW, the tank water was further diluted to 5% and 2.5% for 1 day each.

### Statistics and reproducibility

The otolith data are representative of six, four and six individuals of *G. melas, A. finis and H. mentalis*, respectively. The bootstrap values are shown next to each branch point of the phylogenetic trees. The Fads2 and Elovl5 enzymes were functionally characterised by using coding sequence of the enzyme gene isolated from single individual of each species as representative. The PCR amplifications of genomic *fads2* were performed with template genomic DNA purified from three individuals of *H. mentalis* and all of them showed identical amplification patterns.

### Reporting summary

Further information on research design is available in the Nature Research Reporting Summary linked to this article.

## Supplementary information

Supplementary Information

Description of Additional Supplementary Files

Supplementary Data 1

Supplementary Data 2

Supplementary Data 3

Reporting Summary

## Data Availability

All datasets generated during and/or analysed during the current study are available from the corresponding author on reasonable request. Sequence data that support the finding of this study have been deposited in DDBJ with the accession codes LC487290-LC487298 and the accession codes of all sequence data used in this study are listed in Supplementary Data 1.
