# Supplementary material for: Flatfishes colonised freshwater environments by acquisition of various DHA biosynthetic pathways
Source: Commun Biol. 2020 Sep 18;3:516. doi: 10.1038/s42003-020-01242-3 (PMC7501227; doi:10.1038/s42003-020-01242-3)
Supplement: Supplementary file 1 — Supplementary Information [file 42003_2020_1242_MOESM1_ESM.pdf]

Supplementary Information

Flatfishes colonised freshwater environments by acquisition of various DHA biosynthetic pathways

Yoshiyuki Matsushita, Kaho Miyoshi, Naoki Kabeya,
Shuwa Sanada, Ryosuke Yazawa, Yutaka Haga,
Shuichi Satoh, Yoji Yamamoto, Carlos Augusto Strüssmann,
John Adam Luckenbach, Goro Yoshizaki*

Supplementary Fig. 1–8
Supplementary Table 1

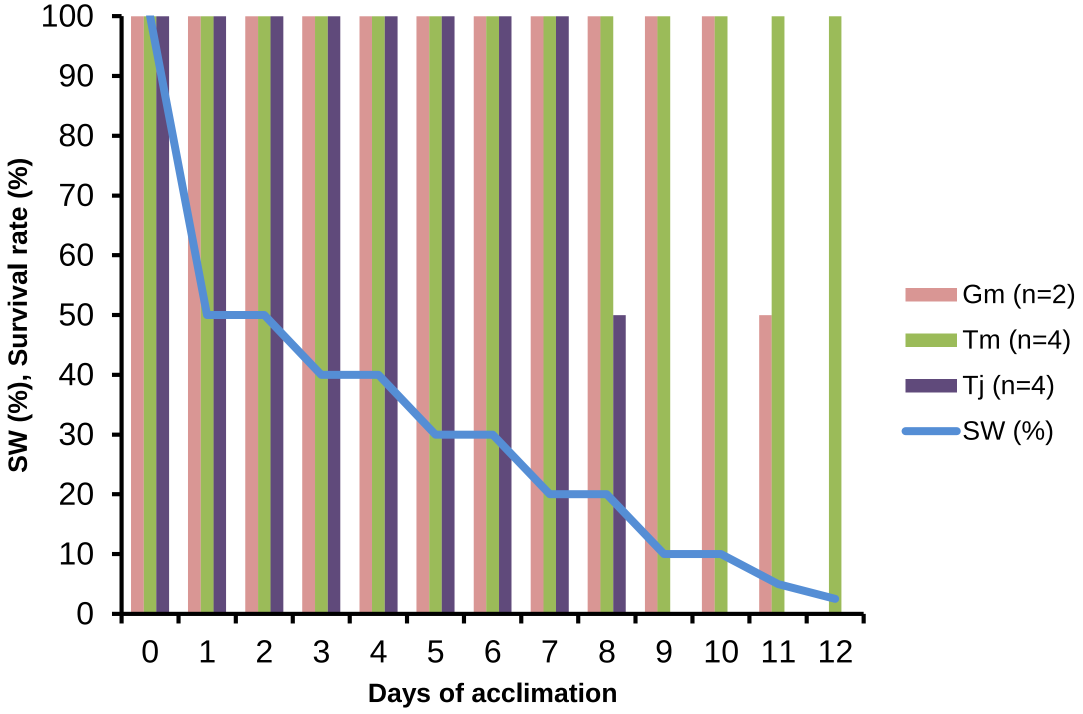

Supplementary Fig. 1 │ Salinity tolerance of *G. melas* relative to other euryhaline and stenohaline species

The dilution level of seawater (SW, %) and survival rate (%) of each species examined are represented by the blue line and the color of each bar, respectively. Gm: *Gymnachirus melas* (pink), Tm: *Trinectes maculatus* (green), Tj: *Trachurus japonicus* (purple).

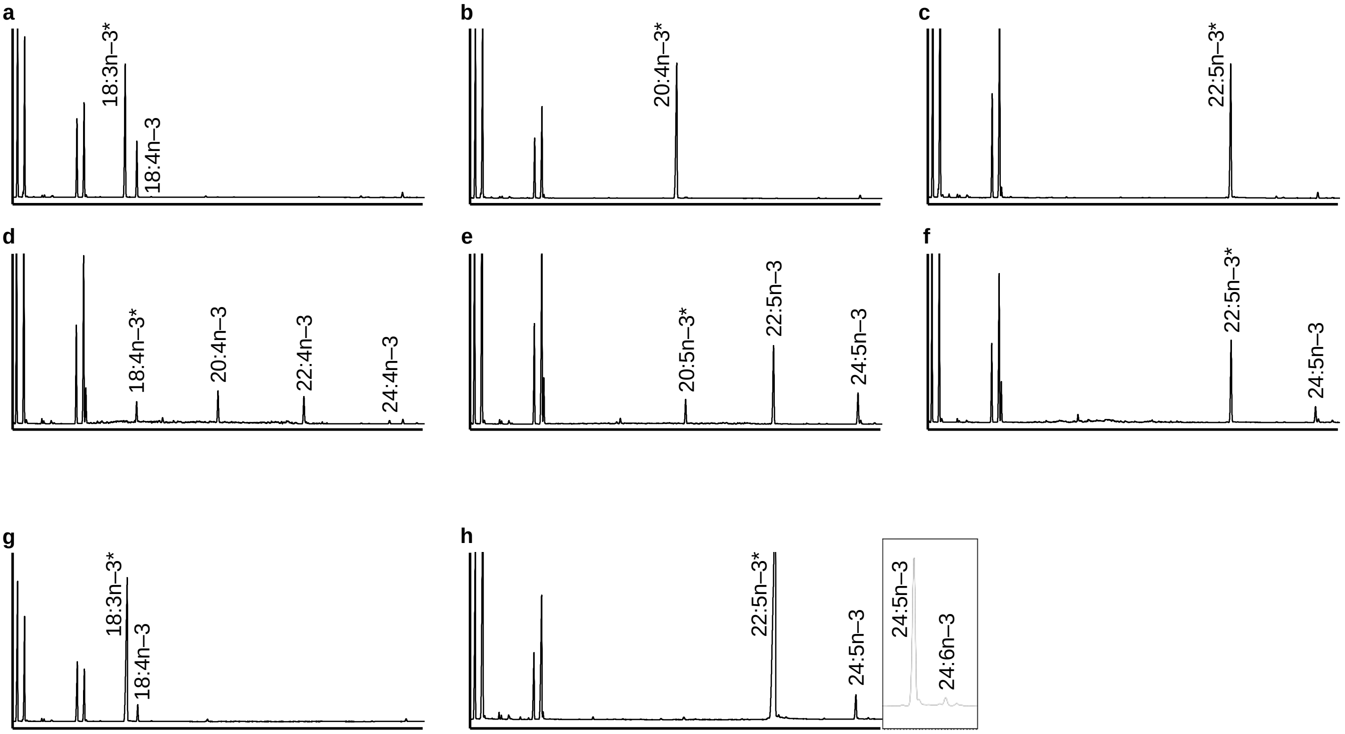

Supplementary Fig. 2 │ Gas chromatogram (*G. melas*)

Chromatograms of fatty acid methyl esters (FAMEs) isolated from recombinant yeast expressing *G. melas* Fads2 (**a**-**c**), *G. melas* Elovl5 (**d**-**f**), or *Danio rerio* Elovl2 and *G. melas* Fads2 (**g**, **h**). The yeast were grown with the exogenously added fatty acid substrate indicated by an asterisk: 18:3n–3 (**a**, **g**), 20:4n–3 (**b**), 22:5n–3 (**c**, **f**, **h**), 18:4n–3 (**d**), and 20:5n–3 (**e**). The inset in **h** shows a magnification of the time when 24:5n–3 and 24:6n–3 appeared.

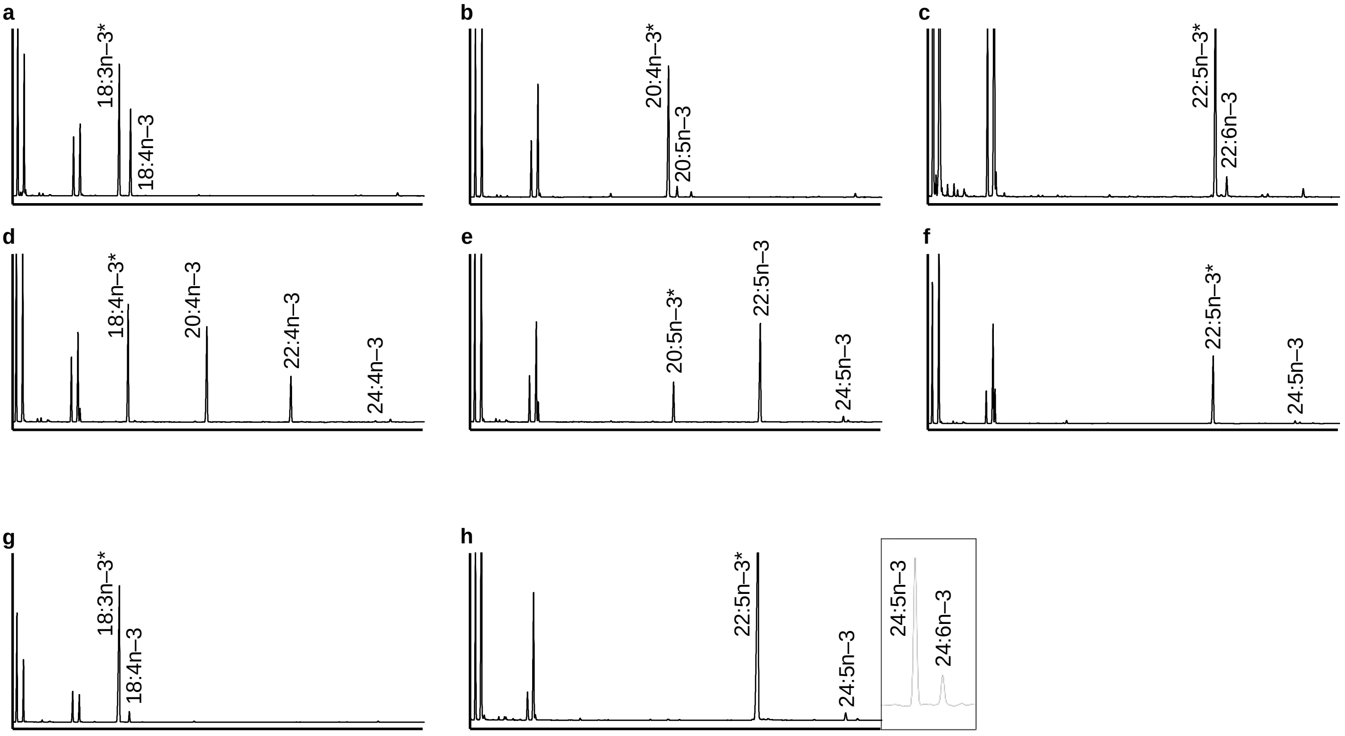

Supplementary Fig. 3 | Gas chromatogram (*T. maculatus*) Chromatograms of fatty acid methyl esters (FAMEs) isolated from recombinant yeast expressing *T. maculatus* Fads2 (**a-c**), *T. maculatus* Elovl5 (**d-f**), or *D. rerio* Elovl2 and *T. maculatus* Fads2 (**g**, **h**). The yeast were grown with the exogenously added fatty acid substrate indicated by an asterisk: 18:3n–3 (**a**, **g**), 20:4n–3 (**b**), 22:5n–3 (**c**, **f**, **h**), 18:4n–3 (**d**), and 20:5n–3 (**e**). The inset in **h** shows a magnification of the time when 24:5n–3 and 24:6n–3 appeared.

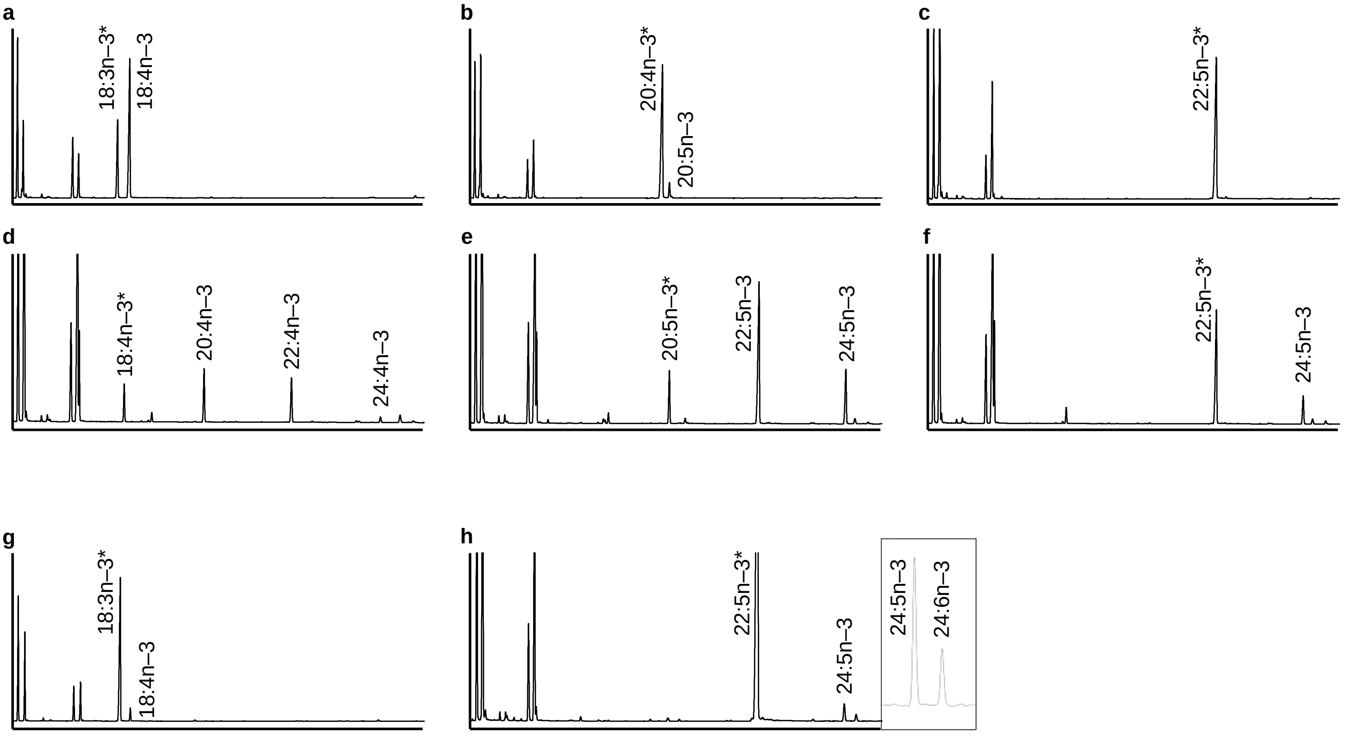

Supplementary Fig. 4 │ Gas chromatogram (*A. finis*)

Chromatograms of fatty acid methyl esters (FAMEs) isolated from recombinant yeast expressing *A. finis* Fads2 (**a-c**), *A. finis* Elovl5 (**d-f**), or *D. rerio* Elovl2 and *A. finis* Fads2 (**g**, **h**). The yeast were grown with the exogenously added fatty acid substrate indicated by an asterisk: 18:3n–3 (**a**, **g**), 20:4n–3 (**b**), 22:5n–3 (**c**, **f**, **h**), 18:4n–3 (**d**), and 20:5n–3 (**e**). The inset in **h** shows a magnification of the time when 24:5n–3 and 24:6n–3 appeared.

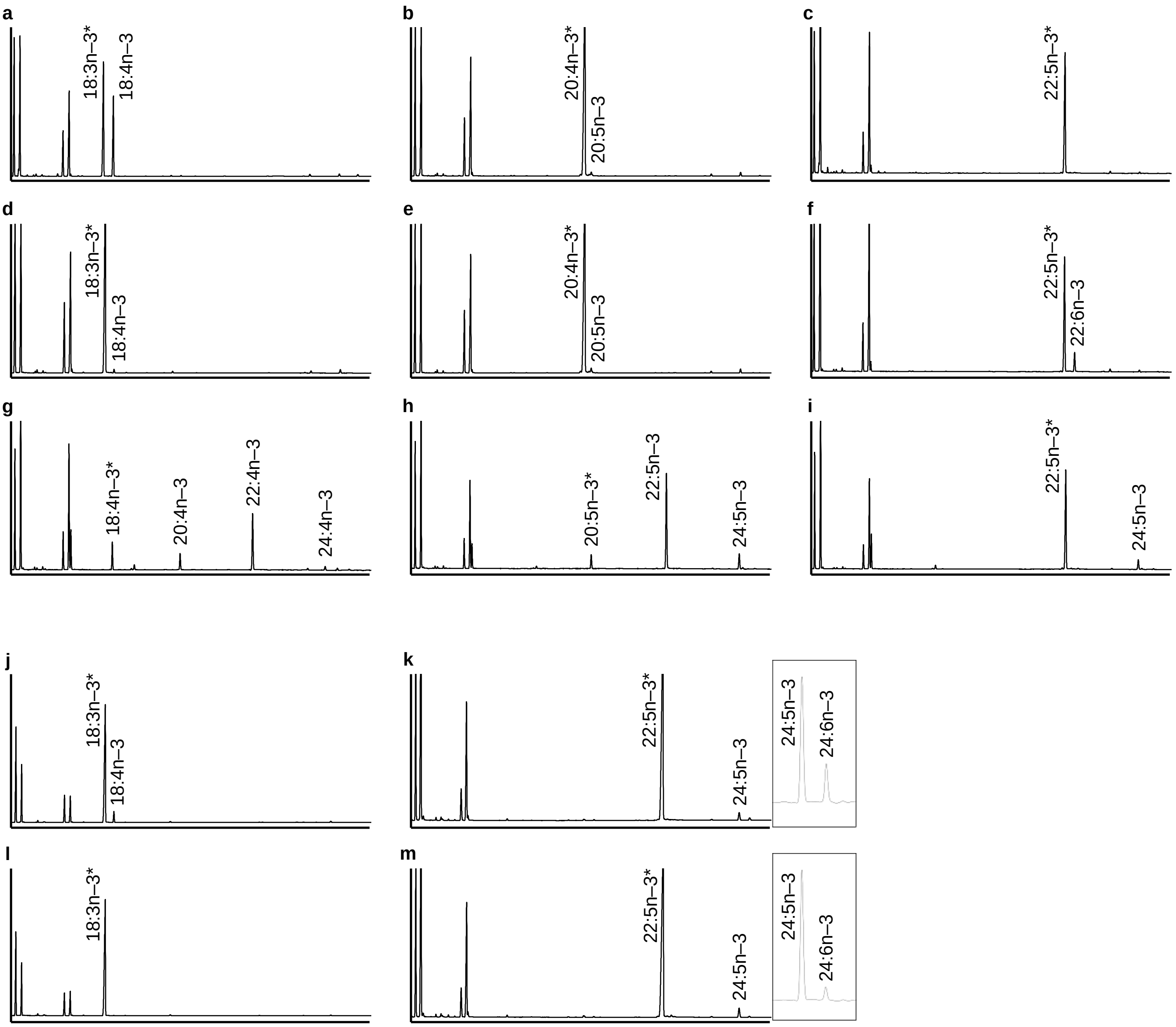

Supplementary Fig. 5 | Gas chromatogram (*H. mentalis*)

Chromatograms of fatty acid methyl esters (FAMEs) isolated from recombinant yeast expressing *H. mentalis* Fads2a (**a-c**), *H. mentalis* Fads2b (**d-f**), *H. mentalis* Elovl5 (**g-i**), *D. rerio* Elovl2 and *H. mentalis* Fads2a (**j**, **k**), or *D. rerio* Elovl2 and *H. mentalis* Fads2b (**l**, **m**). The yeast were grown with the exogenously added fatty acid substrate indicated by an asterisk: 18:3n–3 (**a**, **d**, **j**, **l**), 20:4n–3 (**b, e**), 22:5n–3 (**c**, **f**, **i**, **k**, **m**), 18:4n–3 (**g**), and 20:5n–3 (**h**). The insets in **k** and **m** show a magnification of the times when 24:5n–3 and 24:6n–3 appeared.

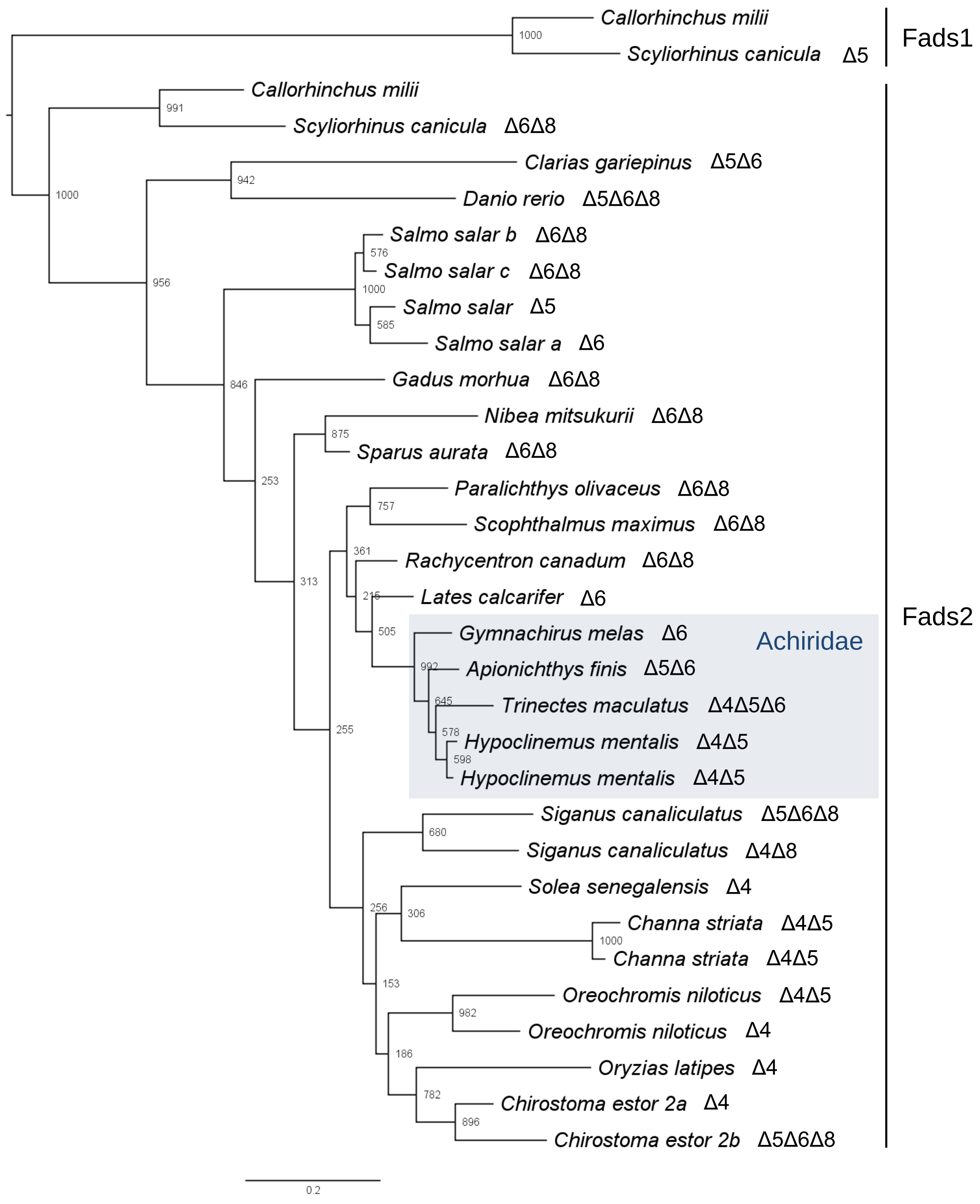

Supplementary Fig. 6 │ Maximum likelihood phylogeny of Fads2 Achiridae Fads2 is highlighted with a blue background. The bootstrap values are shown at the nodes. The functionally characterised Fads2 were shown with their substrate specificities.

```
                                                                          ****
Hm Fads2a  MGGGGQLTESGKQRSGGVYTWEEVQSHDSKNDQWLVVDRKVYNITQWARRHPGGFRVISH  60
Hm Fads2b  ............................................................  60

Hm Fads2a  YAGQDATEAFSAFHPDPAFVQKFLKPLLIGELAASEPSQDRNKNAAIIQDFHTLRTQAEH  120
Hm Fads2b  ........V...................................................  120

                                                                   HXX
Hm Fads2a  KGLFRARPLFFCLHLAHIVLLEALAWLMIWLWGTNWMLTLLCAVMLTISQSQAGWLQHDF  180
Hm Fads2b  ............................................................  180

           XH                                 HXXHH
Hm Fads2a  GHLSVFKKSRWNHLLHKFIIGHLKGASANWWNHRHFQHHAKPNIFRKDPDINMLNIFVVG  240
Hm Fads2b  ..........................................................L.  240

                                                Regio
Hm Fads2a  ETQPVEYGIKKIKHMPYNHQHQYFFLVGPPLLIPIFFHIQIMHTMISRHDWVDLVWSMSY  300
Hm Fads2b  ....................................Y..H.........Y.........F  300

Hm Fads2a  YLRYFSCYIPLYGLFGSVALISFVRFLESHWFVWVTQMNHLPMDIDYEKHQDWLTMQLQA  360
Hm Fads2b  ............................................................  360

                               QXXHH
Hm Fads2a  TCNVEQSAFNDWFSGHLNFQIEHHLFPTMPRHNYHLVAPQVRALCAKYGIPYQVKTLSQG  420
Hm Fads2b  .........................................................W..  420

Hm Fads2a  FTDIVRSLKNSGDLWLDAYLHK  442
Hm Fads2b  .A....................  442
```

Supplementary Fig. 7 | Alignment of deduced amino acid sequences of two *H. mentalis* Fads2 proteins

Identical residues are indicated with dots and shaded in black. The heme-binding motif (HPGG) and three histidine boxes (HXXXH, HXXHH, and QXXHH) are conserved in the Fads family. The motif related to substrate specificity, or regioselectivity (Regio), is also indicated.

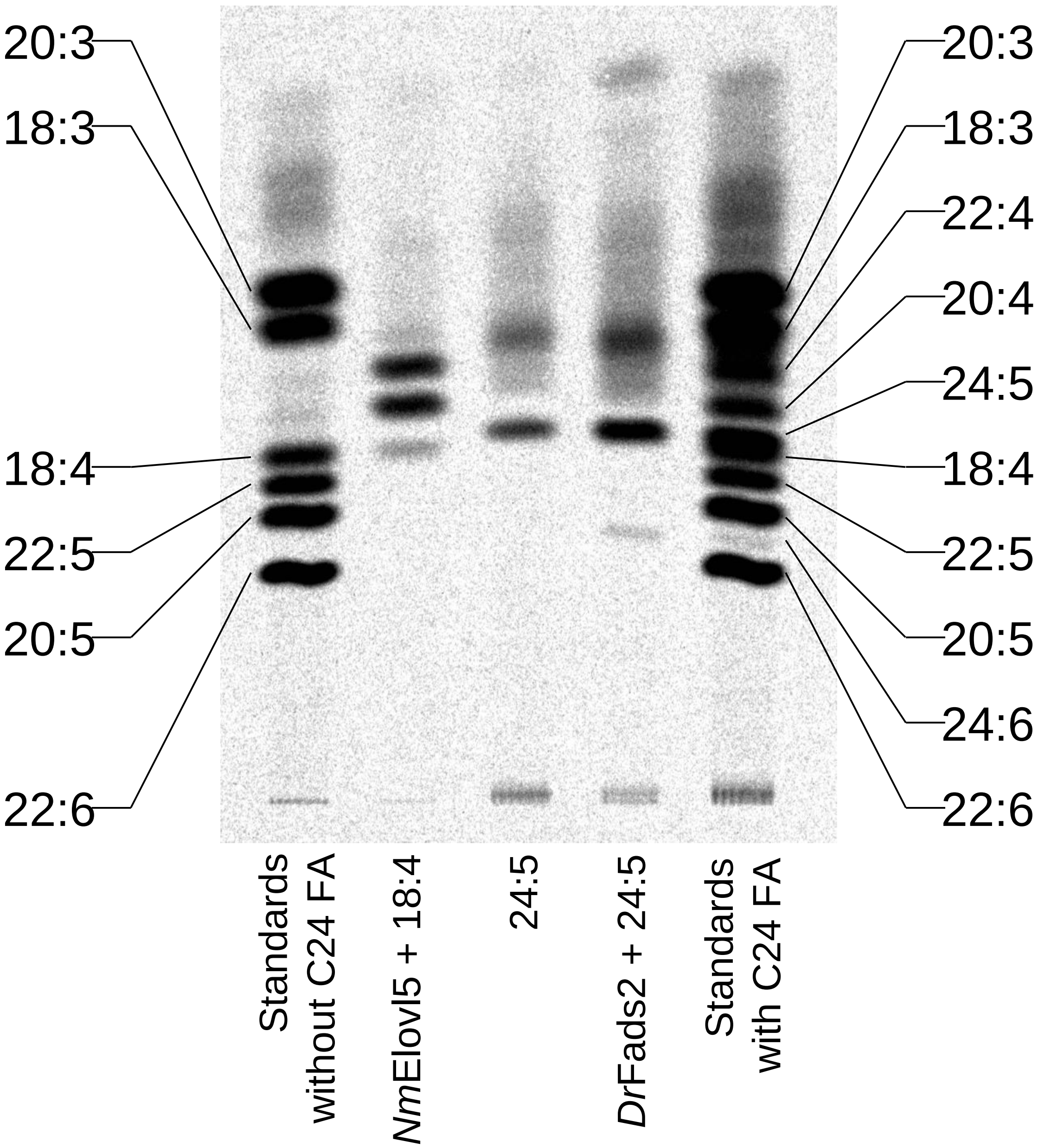

Supplementary Fig. 8 │ Positions of 24:5n–3 and 24:6n–3 on TLC plate

Autoradiography of TLC plate-developed radiolabelled fatty acid methyl esters (FAMEs). Standards for 20:4n–3 and 24:6n–3 were biosynthesised from 18:4n–3 and 24:5n–3 by the yeast transformed with pYES2 carrying *elovl5* from *Nibea mitsukurii* and *fads2* from *D. rerio*, respectively, and confirmed their positions by developing along with the other FAME standards.

Supplementary Table 1 │ Desaturase and elongase activities shown by fatty acid-metabolising enzymes of Achiridae expressed in the recombinant yeast

Supplementary Table 1a. Conversion rates of Elovl5 (%)

Substrate	Product	*G. melas*	*T. maculatus*	*A. finis*	*H. mentalis*	Activity
18:4n–3	20:4n–3	44.8	48.2	37.5	14.9	C18→20
	22:4n–3	31.1	32.8	34.2	60.6	C20→22
	24:4n–3	3.8	3.3	4.5	3.8	C22→24
20:5n–3	22:5n–3	59.5	73.8	62.1	77.6	C20→22
	24:5n–3	23.7	5.4	21.1	11.4	C22→24
22:5n–3	24:5n–3	14.2	3.8	17.7	5.9	C22→24

Supplementary Table 1b. Conversion rates of Fads2 (%)

Substrate	Product	*G. melas*	*T. maculatus*	*A. finis*	*H. mentalis*		Activity
					Fads2a	Fads2b	
18:3n–3	18:4n–3	26.2	39.8	67.6	40.5	1.3	Δ6
20:4n–3	20:5n–3	0.6	6.5	7.3	2.0	2.3	Δ5
22:5n–3	22:6n–3	n.d.	7.2	1.1	n.d.	14.0	Δ4

Supplementary Table 1c. Conversion rates of Fads2 toward 24:5 using co-expression vector (%)

Fads2		Control→Product	24:5n-3→24:6n-3	$\Delta_{24:5n\text{-}3}/\Delta_{control}$
G. melas		7.1	4.9	0.7
T. Maculatus		5.3	16.4	3.1
A. finis		6.2	27.8	4.5
H. mentalis	a	6.3	23.4	3.7
	b	n.d.	8.8	-

Supplementary Table 1d. Conversion rates of Fads2 mutants (%)

mutant	18:3n-3→18:4n-3 (Δ6 activity)	22:5n-3→22:6n-3 (Δ4 activity)	Δ4/Δ6
Fads2b V69A	0.7	5.7	8.2
Fads2b L239V	1.1	4.8	4.5
Fads2b Y277F	23.8	2.3	0.1
Fads2b H280Q	3.2	5.6	1.8
Fads2b Y290D	1.3	4.0	3.0
Fads2b F300Y	0.6	1.8	3.1
Fads2b W418S	0.9	4.9	5.3
Fads2b A422T	0.7	4.0	5.7
Fads2a F277Y	2.6	n.d.	-
Fads2a F277Y+Q280H	0.6	1.2	1.9
