# Supplementary material for: Flatfishes colonised freshwater environments by acquisition of various DHA biosynthetic pathways
Source: Commun Biol. 2020 Sep 18;3:516. doi: 10.1038/s42003-020-01242-3 (PMC7501227; doi:10.1038/s42003-020-01242-3)
Supplement: Supplementary file 2 — Description of Additional Supplementary Files [file 42003_2020_1242_MOESM2_ESM.docx]

Description of Additional Supplementary Materials for

**Flatfishes colonised freshwater environments by acquisition of various DHA biosynthetic pathways**

Yoshiyuki Matsushita, KahoMiyoshi, Naoki Kabeya, Shuwa Sanada, Ryosuke Yazawa, Yutaka Haga, Shuichi Satoh, Yoji Yamamoto, Carlos Augusto Strüssmann, John Adam Luckenbach, Goro Yoshizaki

File Name: Supplementary Data 1

Description: Reference sequences with accession numbers

File Name: Supplementary Data 2

Description: Primers

File Name: Supplementary Data 3

Description: PCRconditions
